# Microencapsulated Extracts From Banana Flowers Induce Milk Production in Lactating Rats Through Increased α‐Lactalbumin, Aquaporin, and Prolactin Levels

**DOI:** 10.1155/sci5/5148782

**Published:** 2026-03-07

**Authors:** Acharaporn Issuriya, Palika Wetchakul, Thammarat Kaewmanee, Surasak Limsuwan, Jo Aan Goon, Sineenart Sanpinit

**Affiliations:** ^1^ Division of Health and Applied Sciences, Faculty of Science, Prince of Songkla University, Hat Yai, Songkhla, 90110, Thailand, psu.ac.th; ^2^ Department of Applied Thai Traditional Medicine, School of Medicine, Walailak University, Thasala, Nakhon Si Thammarat, 80160, Thailand, wu.ac.th; ^3^ Center of Excellence in Tropical Pathobiology, Walailak University, Tasala, Nakhon Si Tammarat, 80160, Thailand, wu.ac.th; ^4^ Department of Food Science and Nutrition, Faculty of Science and Technology, Prince of Songkla University, Pattani, 94000, Thailand, psu.ac.th; ^5^ Traditional Thai Medical Research and Innovation Center, Faculty of Traditional Thai Medicine, Prince of Songkla University, Hat Yai, Songkhla, 90110, Thailand, psu.ac.th; ^6^ Center of Antimicrobial Biomaterial Innovation-Southeast Asia and Natural Product Research Center of Excellence, Faculty of Science, Prince of Songkla University, Hat Yai, Songkhla, Thailand, psu.ac.th; ^7^ Department of Biochemistry, Faculty of Medicine, Universiti Kebangsaan Malaysia, Kuala Lumpur, 56000, Malaysia, ukm.my

**Keywords:** aquaporins, banana blossom, breast milk production, galactagogue activity, herbal, *musa x paradisiaca*

## Abstract

*Musa sapientum* L., commonly known as the banana flower (BF), is used as a galactagogue in Thai traditional medicine. This study aimed to assess the galactagogue potential of microencapsulated extracts from the BF and its impact on serum prolactin level, α‐lactalbumin (LALBA), and aquaporin (AQP) protein levels in the mammary glands of lactating rats. Milk production was determined by measuring pup weight during the suckling period. The control group was administered distilled water orally, whereas Group II was administered domperidone at doses of 2.7 mg/kg. Groups III and IV were orally administered BF at doses of 250 and 500 mg/kg, respectively, from the 3^rd^ day to the 12^th^ day postpartum. On the 12^th^ day, blood samples and mammary gland tissues were collected for analysis. Protein levels of AQP‐1, AQP‐3, AQP‐5, LALBA, and serum prolactin were assessed. Additionally, a histopathological examination of the mammary glands was performed. BF doses of 250 and 500 mg/kg were found to increase milk production, pup weight, serum prolactin levels, and protein levels of AQP‐1, AQP‐3, and AQP‐5 compared to the control group. Transverse sections of the mammary glands from rats treated with 250 and 500 mg/kg exhibited a marked increase in milk secretion within the alveoli. These findings suggest that BF possesses significant galactagogue activity.

## 1. Introduction

Postpartum hypogalactia (PH) is a frequently encountered condition that significantly affects growth and development in infants. It is defined as the presence of minimal or no breast milk production throughout the lactation phase [1]. The World Health Organization (WHO) advises exclusive breastfeeding for infants in the initial 6 months. However, global statistics indicate that only 38% of infants under six months of age are exclusively breastfed, falling short of the target of 50% by 2025 [2]. According to a recent UNICEF‐supported survey in 2023, almost one in three mothers (29%) in Thailand chose exclusive breastfeeding for the first six months of their babies’ lives, showing a substantial increase from 14% in 2019. However, despite this improvement, the rate still falls considerably short of the global nutritional target of 50% by 2025 [3].

The mechanisms involved in milk production include the activities of aquaporins (AQPs), alpha‐lactalbumin (LALBA), and prolactin. AQPs are integral membrane proteins that serve essential functions in facilitating the movement of water across cellular membranes and regulating water balance within the body. The transport of water through the circulatory system and its movement to the mammary glands are pivotal processes in milk synthesis and secretion. AQP1 is primarily expressed in capillaries, whereas AQP3 and AQP5 are predominantly expressed in epithelial cells and ducts [4]. LALBA constitutes the primary protein in breast milk, accounting for 20%–25% of the total protein content, and is known to serve various physiological functions during the neonatal period. Within the mammary gland, it plays a role in lactose synthesis, consequently aiding milk production and secretion by establishing an osmotic “drag” [5]. Prolactin is involved in numerous physiological processes, with its main role being the stimulation of milk production and the development of mammary glands in mammary gland tissue. Specifically, it supports the growth of mammary alveoli and structures within the mammary glands that are responsible for milk production [6]. Enhancing breastfeeding methods, such as promoting the baby’s correct sucking reflex and starting breastfeeding early, are frequently advised to alleviate PH. Occasionally, pharmacological treatments, such as metoclopramide, oxytocin, and domperidone, are employed. However, safety concerns have restricted their use [7]. Herbal galactagogues are increasingly used as an alternative method to boost breast milk production. In Thailand, various herbal formula galactagogues are widely used to boost breast milk production including formulas, such as Ya‐Pra‐Sa‐Naam‐Nom [8]. Plook‐Fire‐Thatu [9], Tri‐Than‐Thip [10], and Wang Nam Yen herbal tea [11]. Moreover, traditional Thai herbal galactagogues, such as *Piper nigrum*, *Syzygium aromaticum*, *Zingiber officinale*, *Euphorbia hirta*, *Allium tuberosum*, *Carica papaya*, and *Musa sapientum*, are significantly correlated with increased breastmilk production [12].

*Musa sapientum* L. or *Musa x paradisiaca* or banana, known in Thai as Kluai “Namwa,” is a widely recognized plant found across Thailand and frequently employed to boost milk production in breastfeeding mothers without any notable adverse effects [13]. Banana plants are used to enhance milk production. However, its taste can be slightly bitter, particularly in less tender areas, which may pose challenges for consumption. Therefore, further research is required to explore the development of new banana flower (BF) forms. This study aimed to investigate the in vivo activity of microencapsulated extracts from BF on milk production; protein levels of AQP‐1, AQP‐3, AQP‐5, and LALBA; serum prolactin levels; and the histopathology of mammary glands in lactating Wistar rats.

## 2. Methods

### 2.1. Microencapsulated Extracts From BF Preparation

In this study, the BFs used were 30–35 days old or 4–5 weeks old. The BFs were cleaned with distilled water, and the banana bracts were removed until the flowers appeared white. Next, cut the BFs into small pieces, dry them at 60°C for 5–6 h, and grind them into a fine powder using a grinder. Then, sift the powder through a 60‐mesh sieve to obtain particles smaller than 250 μm.

The BF extract was obtained from powder using 95% ethanol as a solvent, with a sample to solvent ratio of 1:20 (weight to volume). The extraction process utilized ultrasound‐assisted extraction (BXMZDX51‐T model, 20 kHz, 100 W) under the following conditions: a temperature of 70°C for 60 min. The total soluble solid content of the extract was measured at 1.64% w/v. This BF extract was then used to prepare the microencapsulated extracts.

Microencapsules were prepared using the spray‐drying technique with the SD‐06 model (Lab Plant, UK). The BF extract was used as the core material, and maltodextrin (DE 10–12) and gum arabic were used as the coating materials. The ratio of the core material to the encapsulating material was calculated to be 1:10 (w/w solid), with encapsulant ratios of maltodextrin to gum arabic at 10:0, 8:2, and 6:4. The core and encapsulating solution was prepared with deionized (DI) water to a concentration of 5% w/v and mixed thoroughly. The mixture was then homogenized at 13,000 rpm for 10 min and dried using a spray dryer. The spray dryer settings were an inlet temperature of 180 ± 2°C, an outlet temperature of 90 ± 2°C, pump setting of 7, fan setting of 30, and Deb locker Fast 500 mL per 90 min.

### 2.2. Animals and Experimental Protocol

Female *Rattus norvegicus* and Wistar rats (aged 6–7 weeks and 14–16 days pregnant) were purchased from Nomura Siam International Co., Ltd. (Bangkok, Thailand). They were kept in standard laboratory conditions (temperature: 22 ± 2°C and relative humidity: 55 ± 5%), with a 12‐h light–dark cycle, and had unrestricted access to food and water (ad libitum) during the acclimation period. All animal care procedures and experiments were approved by the Animal Ethics Committee of Walailak University, Thailand. Approval was obtained using reference number WU‐ACUC‐65070.

### 2.3. Galactagogue Activity of the Microencapsulated Extracts From BF

Three days after parturition, twenty‐four lactating rats (dams) each of which had twelve pups were divided into four groups (*n* = 6). The control group of rats (Group I) was administered distilled water; the positive control group (Group II) was administered domperidone 2.7 mg/kg/day; and Groups III and IV were treated with 250 and 500 mg/kg/day BF, respectively. All substances were orally administered to the dams from day three to day 12 of parturition. Every day during the study period, 18 h after the treatment of dams, that is to say, 2.00 pm the day before, all pups were weighed daily at 9.00 am (W1), removed from their mothers, and starved for 4h. At 1.00 pm, the pups were weighed (W2) and returned to their mothers for a one‐hour breastfeeding period. They were then weighed again (W3) at 2.00 pm. According to this method, milk yield was determined by comparing the weight of the pups before and after the feeding sessions. Any increase in weight was attributed to milk ingested by the pups. However, it is important to note that during each feeding session, some of the ingested milk was utilized for energy production, supporting respiration, and pup movement. Consequently, the assessment of milk yield involves the evaluation of the correlation coefficient of weight loss. The measured milk production was calculated using the following equation [14]:

(1) Daily milk production gday=W32−W+W21−W4.

The expression (W3‐W2) represents the weight gain of the pups after lactation, whereas (W2‐W1)/4 denotes the correlation coefficient of weight loss. The daily weight of the pups was denoted as W1.

### 2.4. Measurement of Mammary Gland Parameters

Following the final weighing pup on day 12 of lactation, all the dams were weighed and euthanized at the end of the experimental period by intraperitoneal administration of sodium thiopental at a dose of 150 mg/kg. Blood samples were collected by cardiac puncture. Following the dissection of the mother rats, the six pairs of mammary glands (1: cervical; 2: anterior thoracic; 3: pectoral thoracic; 4: abdominal; 5: anterior inguinal; and 6: posterior inguinal) on both sides were grossly examined, rapidly removed, and weighed. Equal portions of each mammary gland were removed and placed in 10% neutral‐buffered formalin for histopathological analysis. The remaining mammary gland samples were frozen and stored at −80°C for enzyme‐linked immunosorbent assay (ELISA). Mammary Gland Parameters: The mammary gland index weight (I.W.) was computed following Matousek’s method (1969) and expressed as follows:

(2) I.W.=mammary gland weight g/body weight g∗100.

### 2.5. Determination of Serum Levels of Lactogenic Hormones as Prolactin

Three milliliters of blood was collected from each rat into a serum clot activator tube. Blood was placed on a laboratory bench for 20 min in a slanted position to allow coagulation. Subsequently, blood samples were centrifuged at 3000 × *g* for 10 min. Serum was harvested, and prolactin levels were evaluated using the ADVIA Centaur XP Immunoassay System (Germany).

### 2.6. Determination of AQP‐1, AQP‐3, AQP‐5, and LALBA by ELISA Assay

The mammary gland tissues were homogenized in ice–cold PBS (pH 7.4), and the supernatant was removed. The levels of AQP‐1, AQP‐3, AQP‐5, and LALBA were quantified using an ELISA following the manufacturer’s protocol provided by Sunlong Biotech, Zhejiang, China.

### 2.7. Histological Study of Mammary Gland by Hematoxylin and Eosin Staining

The thoracic mammary gland tissues were fixed in 10% neutral‐buffered formalin and embedded in paraffin. Paraffin sections of 5 μm thickness were prepared using a rotary microtome and stained with hematoxylin and eosin (H&E). The slides were examined and imaged using a Canon camera (16 MG) linked to an optical microscope at 10X and 40X magnifications (Olympus Inc., Tokyo, Japan). Histological evaluation focused on alveolar development, lumen size, and epithelial cell morphology.

### 2.8. Statistical Analysis

The data were presented as mean ± standard error of means (SEM). Statistical analyses were conducted using one‐way analysis of variance (ANOVA), and differences between means were assessed using Duncan’s multiple comparison test; all analyses were performed using SPSS Version 20 (SPSS Inc., Chicago, IL, USA). Statistical significance was set at *p* < 0.05.

## 3. Results

### 3.1. Effects of the Microencapsulated Extracts From the BF on Milk Production

Daily and total milk production are presented in Table 1. Maternal rats treated with domperidone and BF doses of 250 and 500 mg/kg exhibited significantly higher (*p* < 0.05) milk production per day than the control group, which is consistent with the total milk production results. Throughout the 10‐day lactation period, total milk production in maternal rats treated with BF doses of 250 and 500 mg/kg was significantly higher (*p* < 0.05) than that in the control group. Similarly, total milk production in the domperidone group was significantly higher than that in the control group. However, among the maternal rats, those administered with BF doses of 500 mg/kg demonstrated the highest milk production during the lactation period, followed by those treated with domperidone and BF doses of 250 mg/kg, respectively.

**TABLE 1:** Milk production of all groups during 10 days of lactation.

Treatment	Milk production (g/day)	Total milk production during 10 days (g)
Control	4.28 ± 0.55	214.13 ± 2.78
Domperidone	7.95 ± 1.18^∗^	477.22 ± 7.10^∗^
BF250	7.23 ± 0.84^∗^	434.07 ± 5.10^∗^
BF500	9.16 ± 1.49^∗^	549.84 ± 8.95^∗^

*Note:* The results were obtained from a sample size of *n* = 6 and are presented as mean ± SEM.

^∗^Indicates a statistically significant difference compared with the control group (*p* < 0.05).

Figure 1 illustrates the milk production trends throughout the lactation period and identifies the peak lactation time for all treatment groups (Figure 1(a)). From day five to day 12 post‐treatment, maternal rats administered domperidone and BF at doses of 250 and 500 mg/kg exhibited significantly higher milk production (*p* < 0.05) than the control group. Similarly, mirroring the daily weight gain of the pups from days six to 12 of the lactation period, treatment with domperidone and BF at doses of 250 and 500 mg/kg resulted in a significant increase (*p* < 0.05) compared to the control group (Figure 1(b)).

FIGURE 1The effect of microencapsulated extracts from banana flower (BF) on milk production 18 h after gavage (a) and pup weight (b). Values are expressed as means ± SEM, *n* = 6. Statistically significant differences are given compared to the control group (ANOVA followed by Duncan). ^∗^ *p* < 0.05.

**Figure:**
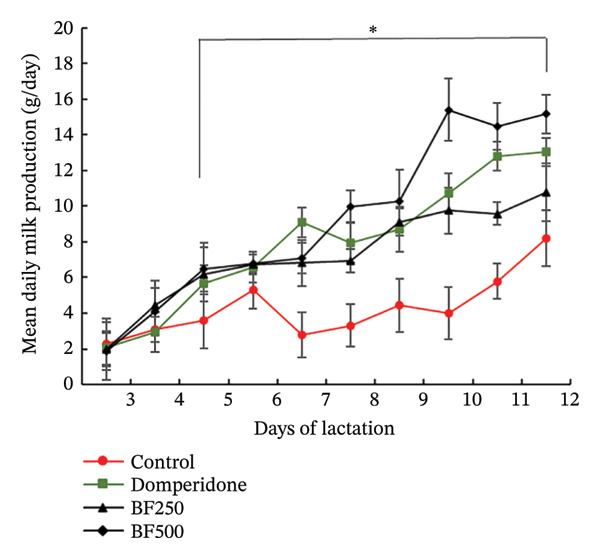
(a)

**Figure:**
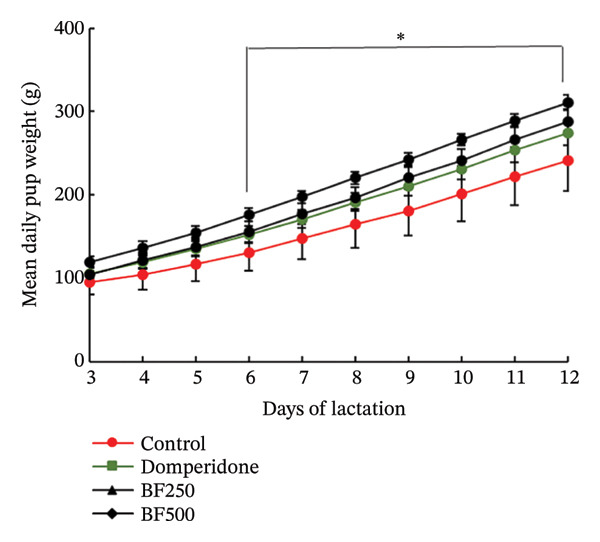
(b)

### 3.2. Effects of the Microencapsulated Extracts From the BF on Mother Weight and Mammary Gland Index Weight

The weights of all maternal rats were recorded daily before each breastfeeding session. No significant differences in maternal weight were observed between the groups during the lactation period, as shown in Figure 2(a). At the end of the experiment, all maternal rats were euthanized and the mammary gland tissue was collected and weighed. Mammary gland index weights are shown in Figure 2(b). The mammary glands of maternal rats treated with domperidone and BF at doses of 250 and 500 mg/kg exhibited significantly higher (*p* < 0.05) mammary gland index weights than those of the control group. Furthermore, the body weights of the maternal rats receiving domperidone and BF at doses of 250 and 500 mg/kg did not differ from those of the control group. However, the mammary gland tissue weight was significantly higher in these groups than in the control group.

FIGURE 2The effect of microencapsulated extracts from banana flower (BF) on mother rat weight for 10 days (a) and mammary gland’s index weight (I.W.) (b). Values are expressed as means ± SEM, *n* = 6. Statistically significant differences are given compared to the control group (ANOVA followed by Duncan). ^∗^ *p* < 0.05.

**Figure:**
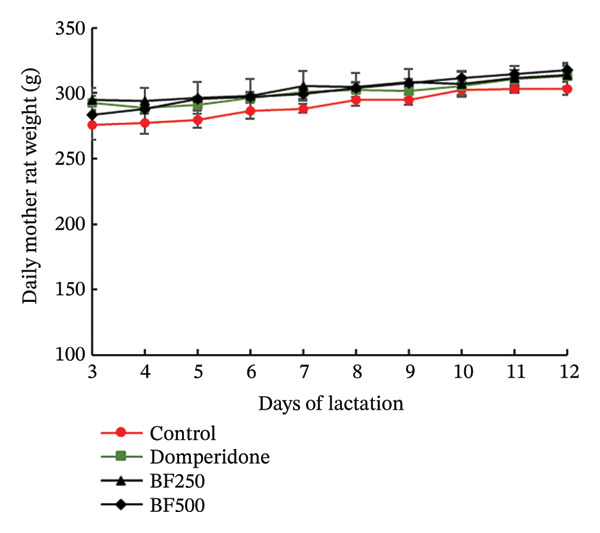
(a)

**Figure:**
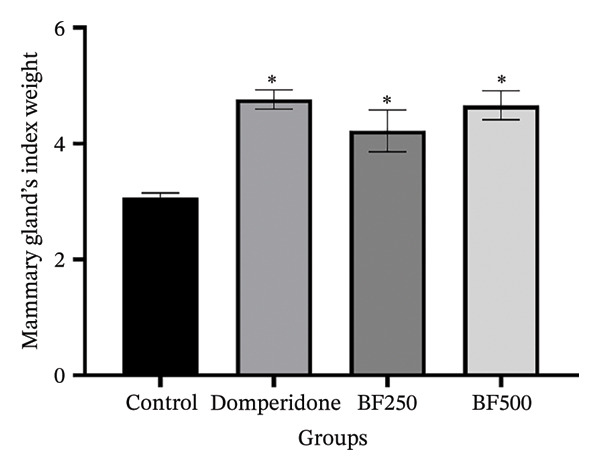
(b)

### 3.3. Effects of the Microencapsulated Extracts From the BF on Prolactin Level in Serum

**FIGURE 3:**
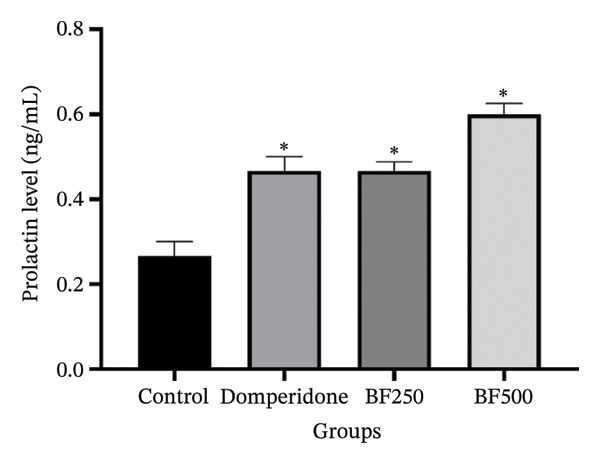
The effect of microencapsulated extracts from banana flower (BF) on prolactin level in serum. Values are expressed as means ± SEM, *n* = 6. Statistically significant differences are given compared to the control group (ANOVA followed by Duncan). ^∗^ *p* < 0.05.

In the control group, the serum prolactin concentration was the lowest at 0.28 ± 0.04 ng/mL. Conversely, in the groups treated with domperidone and BF doses of 250 and 500 mg/kg, the serum prolactin levels were significantly higher (*p* < 0.05), measuring 0.47 ± 0.03, 0.47 ± 0.02, and 0.60 ± 0.03 ng/mL, respectively, compared to the control group (Figure 3).

### 3.4. Effects of the Microencapsulated Extracts From the BF on AQP‐1, AQP‐3, AQP‐5, and LALBA Levels in Mammary Gland Tissue

We used ELISA to quantitatively evaluate the effects of treatment with domperidone and BF doses of 250 and 500 mg/kg on the protein levels of AQPs and LALBA in maternal rats. Significant increases (*p* < 0.05) were observed in the protein levels of AQP‐1, AQP‐3, and AQP‐5 in mammary gland tissue treated with domperidone and BF at doses of 250 and 500 mg/kg compared to the control group. Of note, a BF dose of 500 mg/kg displayed the highest protein levels of AQP‐1 (2.07 ± 0.01 ng/mL) and AQP‐5 (2397.09 ± 181.49 pg/mL), whereas the control group exhibited the lowest protein levels of AQP‐1 and AQP‐5 at approximately 1.53 ± 1.06 ng/mL and 1711.01 ± 59.67 pg/mL, respectively (Figures 4(a), 4(c)). The administration of domperidone at a dose of 2.7 mg/kg resulted in the highest protein levels of AQP‐3 (3272.68 ± 54.14 pg/mL), whereas the control group showed the lowest protein levels of AQP‐3 at 2437.97 ± 44.54 pg/mL (Figure 4(b)).

FIGURE 4The effect of microencapsulated extracts from banana flower (BF) on AQP‐1 (a), AQP‐3 (b), AQP‐5 (c), and LALBA (d) levels in mammary gland tissue. Values are expressed as means ± SEM, *n* = 6. Statistically significant differences are given compared to the control group (ANOVA followed by Duncan). ^∗^ *p* < 0.05.

**Figure:**
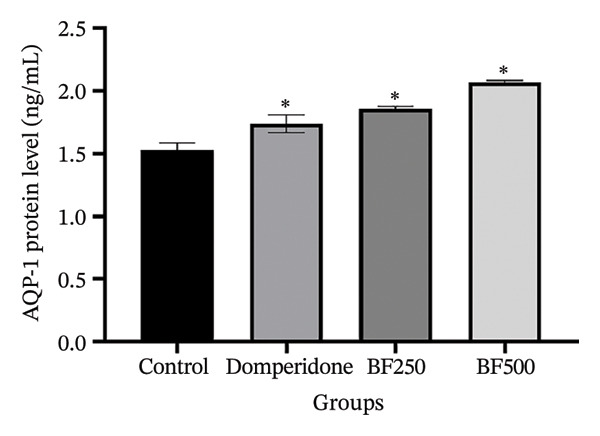
(a)

**Figure:**
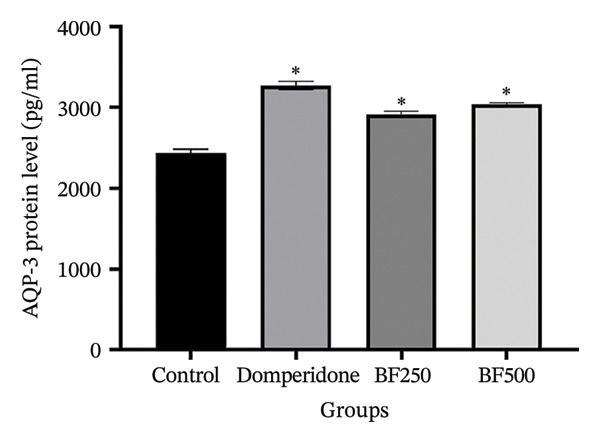
(b)

**Figure:**
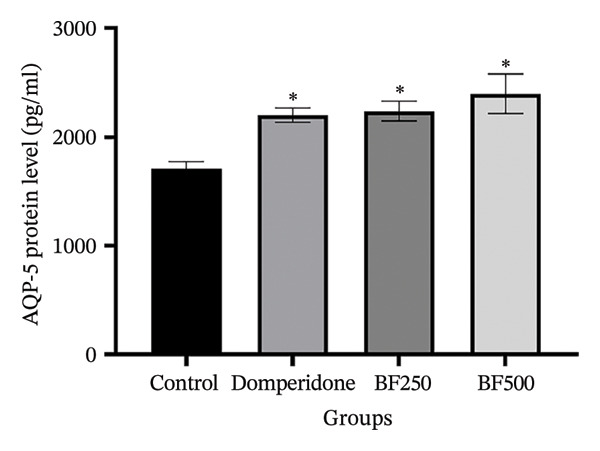
(c)

**Figure:**
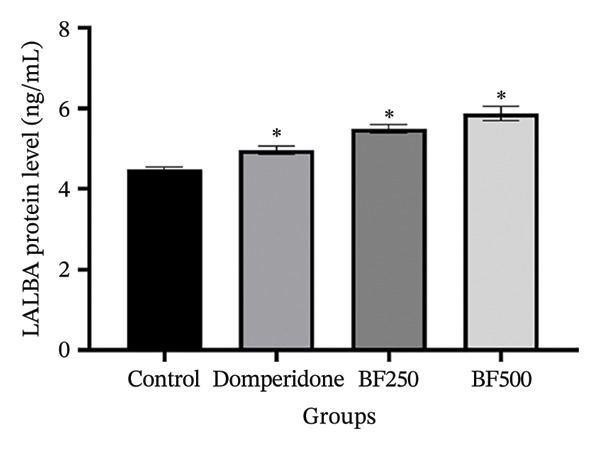
(d)

Figure 4(d) illustrates the protein levels of LALBA in the mammary gland tissue. Mother rats treated with domperidone and BF at doses of 250 and 500 mg/kg exhibited significantly elevated protein levels of LALBA compared to the control group. Among these, a BF dose of 500 mg/kg (5.86 ± 0.18 ng/mL) displayed the highest protein levels of LALBA, followed by a BF dose of 250 mg/kg (5.49 ± 0.11 ng/mL) and domperidone (4.97 ± 0.10 ng/mL), respectively. In contrast, the control group exhibited the lowest protein levels of LALBA at 4.49 ± 0.05 ng/mL.

### 3.5. Effects of the Microencapsulated Extracts From the BF on the Histology of the Mammary Gland

On day 12 of lactation, histological analyses of the mammary glands were conducted. In the control group, alveoli were observed to have little to no milk secretion (as denoted by the letter “*M*” in Figure 5(a)). Conversely, mammary glands treated with domperidone and BF at doses of 250 and 500 mg/kg exhibited fully distended alveoli, prominently displaying abundant signs of milk secretion within the lumina, suggesting active milk production (Figures 5(b), 5(c), 5(d)).

FIGURE 5The effect of microencapsulated extracts from banana flower (BF) on histology of the alveoli in the mammary glands. The control group (a), the domperidone group (b), BF250 group (c), and BF500 group (d). All groups were administered to the lactating rat for 10 days with a sample size of *n* = 6. The letter “*M*” denotes milk. The yellow circle denotes alveoli. The scale bars represent 100 μm for images at 10X and 40X magnification.

**Figure:**
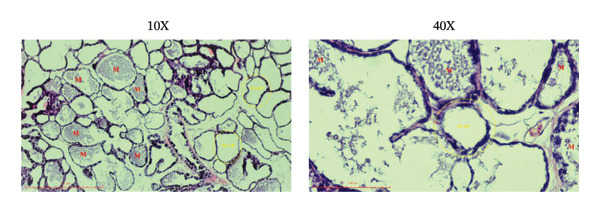
(a)

**Figure:**
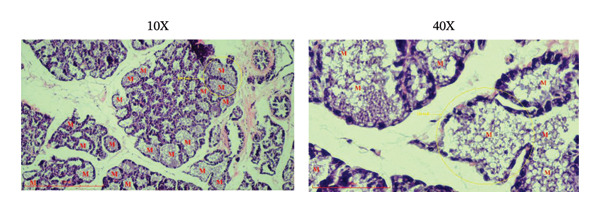
(b)

**Figure:**
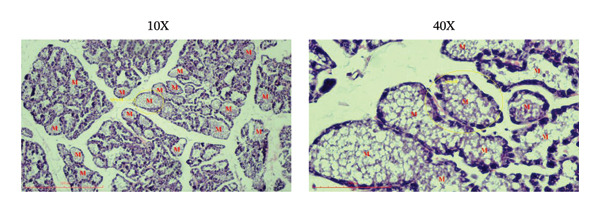
(c)

**Figure:**
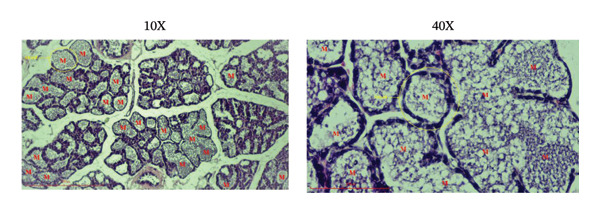
(d)

## 4. Discussion

Measuring the milk production of a maternal rat is typically challenging but can be estimated by observing the weight gain of pups during feeding intervals after separating them from the mother for a period adjusted for any incidental weight loss of the pups [15, 16]. This technique considers pups as efficient suction devices that can drain the mammary glands thoroughly. It is commonly used to assess the lactation‐boosting properties of plant‐based products [7, 15]. This study aimed to evaluate the effects of BF administered at doses of 250 and 500 mg/kg on milk production, protein levels of AQP‐1, AQP‐3, AQP‐5, and LALBA in the mammary glands, and serum prolactin levels in lactating rats. When comparing milk production between treated maternal rats and the control group, it was found that domperidone (2.7 mg/kg), BF (250 mg/kg), and BF (500 mg/kg) significantly increased total milk production. At a dosage of 500 mg/kg, microencapsulated extracts from BF led to total milk production over 10 days that exceeded that of the control group (335.71 g). These outcomes demonstrate superior efficacy compared to those of Mahmood et al. [24], who found that an aqueous extract of the *Musa x paradisiaca* flower at 500 mg/kg resulted in total milk production over 10 days for treated maternal rats, surpassing that of the control group, which was only 39.96 g. This difference may be attributed to several factors, including differences in herbal composition, extraction and formulation methods, and bioavailability. Unlike the aqueous extract used by Mahmood et al., the BF extract in this study was microencapsulated, which may enhance the stability and sustained release of bioactive compounds, thereby improving their biological efficacy. Domperidone significantly increased milk production compared with the control group, confirming its role as a positive galactagogue control. BF administration also significantly enhanced milk production at both tested doses. The BF 250 mg/kg group showed milk production levels comparable to domperidone, whereas the BF 500 mg/kg group exhibited a greater increase in both daily and total milk production than domperidone, indicating a dose‐dependent galactagogue effect of BF. These findings suggest that BF, particularly at the higher dose, may possess galactagogue efficacy comparable to or exceeding that of domperidone, highlighting its potential as a natural alternative for enhancing milk production. The increase in milk production observed in the BF‐treated rats corresponded to an increase in the weight of the mammary gland tissue, suggesting an augmentation in milk production. These findings were consistent with the histological examination results. Our observation that BF‐treated rats exhibited. Lactogenesis is a complex neuroendocrine process that involves the interaction of several physical and emotional factors, along with the action of multiple hormones, mainly prolactin. Prolactin is the most important hormone required for mammary milk production [17–19]. This study demonstrated that BF at a dosage of 500 mg/kg elevated serum prolactin levels by more than twofold compared to those in the control group. This result is similar to that of a previous study by Sani et al. (2019), in which maternal rats treated with the n‐hexane leaf fraction of *Launaea taraxacifolia* demonstrated a threefold increase in serum prolactin compared to the control group, consequently leading to increased milk production. Our current findings indicate that microencapsulated BF extracts at both doses stimulated prolactin secretion, thereby promoting milk production and secretion in maternal rats during lactation. Although an increase in milk supply is beneficial, the quality of milk is significant. LALBA constitutes a significant portion of the whey protein in mammalian milk, including that of humans (approximately 36%), bovines (approximately 17%), and various other species. Prolactin, mediated by STAT5a, enhances the activity of lactose synthase and LALBA activity. It regulates lactose synthesis and has considerable nutritional benefits [21]. In this study, it was observed that microencapsulated extracts from BF, administered at both doses, elevated the serum protein levels of LALBA in maternal rats. This finding suggests that treatment with microencapsulated BF extracts during lactation in rats contributes to enhancing milk quality. While an increase in milk supply is beneficial, the quality of the milk holds significance. LALBA constitutes a significant portion of whey protein in mammalian milk, including human (about 36%), bovine (about 17%), and various other species. Prolactin, mediated by STAT5a, enhances the activity of lactose synthase and LALBA. It plays a role in regulating lactose synthesis and boasts considerable nutritional benefits [21]. In this study, it was observed that microencapsulated extracts from BF, administered at both doses, elevated the serum protein level of LALBA in mother rats. This finding suggests that the treatment with microencapsulated extracts from BF during lactation in rats contributed to enhancing the quality of milk.

In addition to prolactin and LALBA, another determinant of milk secretion is AQP levels. These membrane proteins, which facilitate the movement of water across cell membranes, are expressed in mammary glands, indicating their potential roles in milk secretion [22]. A previous study showed that a polyherbal formula (PHF) containing an extract of *Sauropus androgynus* (L.) Merr., *Trigonella foenum-graceum* L., and *Moringa oleifera* Lam. has the potential to induce galactagogue activity during the lactation period by upregulating the AQP‐1, AQP‐3, and AQP‐5 genes at the mRNA level^4^. Similarly, in line with the findings of Liu et al. [23], we observed that a herbal galactagogue mixture enhanced milk secretion by regulating the protein expression and functionality of AQP‐3 and AQP‐5 in mammary glands. In our study, we discovered that microencapsulated extracts of BF at both doses elevated the protein levels of AQP‐1, AQP‐3, and AQP‐5 in the mammary glands. This suggests that these extracts may have influenced the capillaries of the mammary glands, where AQP‐1 is highly abundant. Furthermore, the microencapsulated BF extracts appeared to affect the epithelial cells and ducts of the mammary glands, where AQP‐3 and AQP‐5 are abundant. Augmented AQP levels in mammary gland tissue lead to increased milk secretion in maternal rats [4, 10, 23].

The microencapsulated BF extract used in this study is known to contain a variety of bioactive secondary metabolites that may contribute to its galactagogue activity. Previous phytochemical screening of *Musa x paradisiaca* (banana) flower extracts has demonstrated the presence of alkaloids, glycosides, steroids, saponins, tannins, flavonoids, and terpenoids in the aqueous and crude extracts, many of which have been implicated in modulating lactation‐related processes [24]. Among these compounds, saponins and tannins are widely reported to enhance prolactin secretion and support milk production, possibly through endocrine modulation and improvements in mammary gland function [25]. Flavonoids and phenolic compounds found in BF extracts also possess significant antioxidants and anti‐inflammatory properties, which may protect mammary tissue integrity and support cellular processes necessary for sustained milk synthesis [26]. Additionally, phytosterols, such as β‐sitosterol identified in BF extracts, could exert hormonal‐like effects and contribute to lactation enhancement via interactions with steroid hormone pathways [24]. Taken together, these phytochemicals may act synergistically to support lactation by stimulating prolactin release, enhancing mammary gland development, and reducing oxidative stress, which collectively could explain the increased milk production observed in BF‐treated animals.

In this study, microencapsulated BF extracts at doses of 250 and 500 mg/kg had comparable effects on milk production during lactation. However, the 500 mg/kg dose demonstrated a greater increase in milk production than the 250 mg/kg dose, as supported by histological analyses. Therefore, the optimal dose for stimulating breast milk production was 500 mg/kg.

## 5. Conclusions

In summary, our findings indicate that BF (250 and 500 mg/kg) substantially boosts milk production. Furthermore, both doses of BF notably increased serum prolactin and AQP‐1, AQP‐3, AQP‐5, and LALBA levels in lactating rat mammary glands. This suggests that herbal galactagogues enhance breast milk production by modulating prolactin, LALBA, and AQPs.

## Author Contributions

Acharaporn Issuriya: conceptualization, methodology, validation, formal analysis, resources, data curation, writing–review and editing, supervision, project administration, and funding acquisition. Sineenart Sanpinit and Palika Wetchakul: conceptualization, methodology, software, validation, formal analysis, investigation, resources, data curation, writing–original draft, writing–review and editing, visualization, supervision, project administration, and funding acquisition. Thammarat Kaewmanee: conceptualization, methodology, formal analysis, resources, writing–review and editing, supervision, and funding acquisition. Surasak Limsuwan: funding acquisition. Jo Aan Goon: writing–original draft and writing–review and editing.

## Funding

This research was supported by the National Science, Research, and Innovation Fund (NSRF) and Prince of Songkla University (Grant No. TTM6601212b).

## Disclosure

All authors read and approved the final manuscript. All authors agreed to be accountable for the research presented.

## Ethics Statement

This study was approved and granted by the Ethics Committee (Animal Care Use and Research Ethics Committee) of the Walailak University, Thailand. Approval was obtained using reference number WU‐ACUC‐65070.

## Conflicts of Interest

The authors declare no conflicts of interest.

## Data Availability

The data that support the findings of this study are available from the co‐corresponding author or the corresponding author upon reasonable request.
